# The Utility of an Online Forward Triage Tool During the SARS-CoV-2 Pandemic: Health Care Provider and Health Authority Perspectives

**DOI:** 10.3389/fpubh.2022.845996

**Published:** 2022-07-08

**Authors:** Janet Michel, Tim S. Kilb, Annette Mettler, Martin Müller, Wolf E. Hautz, Stefanie C. Hautz, Thomas C. Sauter

**Affiliations:** Department of Emergency Medicine, Inselspital, University Hospital, University of Bern, Bern, Switzerland

**Keywords:** OFTT utility, health care providers, health authorities, perspectives, COVID-19, pandemic, telehealth in future, perceived barriers and drivers

## Abstract

**Introduction:**

The SARS CoV-2 pandemic poses major challenges not only to patients but also to health care professionals and policy-makers, with rapidly changing, sometimes complex, recommendations, and guidelines to the population. Online forward triage tools (OFTT) got a major boost from the pandemic as they helped with the implementation and monitoring of recommendations.

**Methods:**

A multiphase mixed method sequential explanatory study design was employed. Quantitative data were collected first and informed the qualitative interview guides. Video interviews were held with key informants (health care providers and health authorities) between 2 September and 10 December 2020. Audio-recordings were transcribed verbatim, coded thematically and compared with patient perspectives (framework).

**Objectives:**

To explore the perspectives of health care providers and authorities in Canton Bern on the utility of a COVID-19 OFTT, as well as elicit recommendations for telehealth in future.

**Results:**

The following themes emerged; (i) accessibility (ii) health system burden reduction (iii) utility in preventing onward transmission (iv) utility in allaying fear and anxiety (v) medical decision-making utility (vi) utility as information source (vii) utility in planning and systems thinking. The health care providers and health authorities further provided insights on potential barriers and facilitators of telehealth in future.

**Conclusion:**

Similar to patients, health care providers acknowledge the potential and utility of the COVID-19 OFTT particularly as an information source and in reducing the health system burden. Data privacy, doctor-patient relationship, resistance to change, regulatory, and mandate issues, and lack of systems thinking were revealed as barriers to COVID-19 OFTT utility.

## Background

The SARS-CoV-2 pandemic has affected nearly every country on the face of earth. Minimizing transmission and efforts to reduce the burden on health systems have been achieved in part through telehealth interventions (1–3). Telehealth is defined as the use of electronic information and communication technologies to provide and support health care (4) and has the potential to deliver quality and cost effective health care (5). Governments world-wide have increased spending on telehealth particularly during this SARS-CoV-2 pandemic (5). There are four main types of telehealth (i) synchronous telehealth, where consultation is conducted across distance using a two way communication (ii) remote patient monitoring, where providers record and monitor patient data at a distance (iii) store and forward telemedicine e.g., OFTTs and (iv) mobile health (6).

Lately technology, particularly online forward triage tools (OFTTs) have gained ground (7), and telehealth is now viewed as a significant health and wealth contributor (8). OFTTs may help patients with their health questions, but also support health care providers (with reduction of cross-infection and health system burden) and healthcare authorities (with planning of testing capacities, allocation of resources, monitoring of recommendations, and information dissemination). With increased use of telehealth, it is essential to explore the attitudes of health care professionals as they influence its uptake (9). Attitudes have been linked to the non-adoption, abandonment and failed attempts to scale up or sustain technological innovations at system level (10). Abandonment and non-adoption of telehealth by users have been extensively reported (11). According to an Australian study, clinician acceptance of telemedicine influences uptake, expansion and sustainability (12) and also overcomes low demand, technological problems, lack of resources, and workforce pressure (12). The department of emergency medicine Insel University Hospital Bern, developed this online forward triage tools (OFTT)- coronatest, in an attempt to reduce the burden on the health care system. The tool was designed as a click-through questionnaire that offered recommendations to users that suspected SARS-CoV-2.

The aim of this study was to explore the perspectives of health care providers and authorities in Bern province on the utility of a COVID-19 OFTT (coronatest.ch) as well as elicit recommendations for telehealth in future.

## Methods

### Context and Intervention

The department of emergency medicine at Insel University Hospital Bern, developed a COVID-19 online forward triage tool (OFTT) (7). The OFTT aimed to reduce the health system burden through the provision of testing recommendations to OFTT users, as well as support users in implementing recommendations made by health authorities. In Switzerland, the Federal Office of Public Health (FOPH) is the primary healthcare authority for the whole country, responsible for developing broader guidelines and legislations. The tool was continuously updated in accordance with the fast-changing official (FOPH) guidelines. At the provincial level (canton), the provincial medical office is responsible for the implementation of these. During the initial phase of the Coronavirus pandemic, the FOPH Confederation assumed extended powers under a special pandemic legislation, which also affected implementation. These key informants did not necessarily use the COVID-19 OFTT, but were privy to it as stewards of the health system.

### Study Design Setting and OFTT Description

A multiphase mixed method sequential explanatory study design was employed to assess the effects and utility of the OFTT. In line with sequential explanatory designs, quantitative data, a major component of the study data were collected first. The quantitative results then informed the qualitative interview guides for both, health care providers and authorities. The first manuscript on patient perspectives is currently under review (13). This manuscript will focus on the perspectives of health care providers and health authorities only.

### Study Aim

The aim of this study was to explore the perspectives of health care providers and authorities in Bern province (canton), on the utility of a COVID-19 OFTT (coronatest.ch), as well as elicit recommendations for telehealth in future. The overarching aim of the study was to assess the effects (quantitatively) and utility (qualitatively) of an OFTT in a pandemic context exploring both patient (13), health care provider and health authority perspectives (see Table 1).

**Table 1:** Study design.

**Data type and collection phase**	**Participants**	**Status**
Phase 1 Quantitative data: online Survey	OFTT users including some health care workers	Under review (13)
Phase 2 Qualitative data: interviews-patient perspectives	OFTT users including some health care workers	Under review (13)
Phase 3 Qualitative data: interviews-health care provider and authorities‘ perspectives	Health care provider and Health authorities who did not use the OFTT per se but are stewards of the health system- perceived OFTT utility	Current manuscript

### Research Question

“As a health care provider or health care authority, what did you find useful about the COVID-19 OFTT (coronatest.ch) and what did you find deficient?”

#### Sub-questions

- What role do you foresee telehealth (OFTT and other tools) playing in future?
- What are the potential barriers and facilitators to telehealth (OFTT and other tools)?

### Qualitative Data

The utility of the COVID-19 OFTT matters not only to patients, but also to other key stakeholders particularly, health care providers and health authorities. To capture the perspectives of this group and further explain patient perspectives on OFTT (13), in-depth semi-structured, video interviews were held with five purposefully selected participants who were health care providers and health authorities in Bern, Switzerland.

### Purposeful Sampling

We purposefully sampled key informants from health care providers that responded to our call and consented to take part in the study. We also contacted the responsible health authorities, and one key informant from each administrative level of Switzerland (Canton and Confederation) consented to take part. It is important to note that there is only one cantonal medical office and one FOPH office.

### Sample Size

Experts suggest saturation as central to qualitative sampling (14). We aimed for both rich narratives and theoretical saturation (framework) and achieved this by the 3rd key informant, and interviews were concluded after the 5th key informant (there is only one provincial medical office and one FOPH office) (see Table 2).

**Table 2:** Key informants.

	**Male**	**Female**	**Total**
Key informants	1	4	5
Total	**1**	**4**	**5**

### Data Collection

Video rather than face-to face interviews were held with key informants due to COVID-19 concerns and social distancing recommendations during the interview period. Interviews were held between 2 September and 10 December 2020. An interview guide that was adapted iteratively was used. See Appendix 1 and 2. Two experienced qualitative researchers held interviews with the health care workers and fielded questions in turns. The health care authority interviews were conducted by 3 interviewers, with a professor joining in the two sessions. The interviews lasted for 30–45 min. Two audio recorders were used in each session. All key informants gave both oral and written consent to recording at the beginning of each session.

### Data Analysis

An inductive and deductive data analysis approach was adopted. Audio-recordings were transcribed verbatim, coded thematically and compared with patient perspectives (framework). We utilized a framework analysis, guided by themes from patient perspectives while remaining open to new themes. Data were managed and analyzed with the aid of MAXQDA2018 (VERBI software, Berlin). Narratives on the OFTT utility, potential stumbling blocks and future role of telehealth (OFTT and other tools) were also elicited.

### Measures to Ensure Trustworthiness of Data

We performed an iterative data collection and analysis, continuously adjusting the interview guide to capture emerging themes so as to ensure dependability. The two qualitative researchers kept reflexive journals and debriefed at the end of each interview. A thick description of participants, context and data collection process have been outlined to ensure transferability.

### Ethics Approval

Our study is embedded in an online forward triage tool set up by the Insel University Hospital in a pandemic setting to primarily prevent health system over load. The evaluation of the usefulness of this tool to the health stake holders, patients, health care providers and health authorities was deemed as quality evaluation hence the ethics committee of the province (Canton) of Bern, Switzerland waived the need for full ethical review (Req-2020-00289) on the 23rd of March 2020 and granted us permission to carry out the study.

## Results

### Accessibility

The tool was not widely advertised. The health authorities at cantonal level shared links of the tool of their own accord.

*Different cantons use different tools with different messages. The lack of collaboration and uniformity of tools is a challenge. We can only recommend what the tool (COVID-19 OFTT) should cover but we unfortunately cannot enforce that*.

Key Informant 5

*Different societies are in charge of OFTTs. The ideal thing is to move from cantonal to national OFTTs*.

Key Informant 4

### Socio-Economic Status

Low socio-economic status has been reported as a barrier to telehealth (15). Below is what the participants revealed;

*I can't say that those from a different socio-economic level were then significantly worse informed than others. I think they all more or less knew what COVID-19 was about. It is interesting that it is easier to convince people from a low socio-economic background. Perhaps it is because they readily accept and also respect medical authority*.

Key Informant 2

*Often you then have certain discussions with academics or with people who then somehow have a certain university background and they somehow project onto the medical field and... Yes, they tend to have stronger opinions and it can be difficult to convince them and it takes too much time*.

Key Informant 2

### Health System Burden Reduction

All health care providers revealed that they were inundated with phone calls from patients seeking information on COVID-19 during the first wave. Most of them were relieved when they heard of the coronatest.ch OFTT and went on to share the link on their websites to increase tool accessibility. One key informant revealed the following;

*Telephonic enquiries became a big problem at the beginning of the pandemic. So, we ended up operating two telephone lines in parallel while at the same time always fully booked. Many patients could not reach us, despite two open lines. And that's why we were very happy when the OFTT was developed. We were then able to say: please take a look at our homepage, we have a link. If you meet the test criteria, come and test*.

Key Informant 1

### Utility in Preventing Onward Transmission

Most of the health care providers found the OFTT effective when it came to triaging patients regarding whether to test or not to test, and thus avoid unnecessary contacts with the health system, but also facilitated only the necessary consultations. Concerns were raised however when it came to the triaging of employees as revealed below;

*Exactly. I think that the tool uses very explicit questions, like having cold like symptoms for a day, stay at home. That is okay if it is a patient that cancels an appointment. The story is different when one of the employees has cold like symptoms for a day, may be because she had a bad day. She cannot work from home and that becomes an issue if the tool recommends her to stay at home. That affects me as an employer and that affects the quality of care, we provide*.

Key Informant 3

### Utility of Tool in Allaying Fear and Anxiety

For the patients, the tool utility in this regard emerged as important (13). Health care providers particularly pediatricians, insisted on the importance of physically seeing the child and doing a physical exam. They argued that parents are very different in how well they deal with their own child when it is sick and not doing well. Below is what was said;

*The insurance company assessments, right? These telephonic triages, or the health insurance companies that do telephonic triage? The history is neither good history nor is the assessment sufficient reassurance for the patient. I think that if it's someone you trust, a doctor you've known for a long time, that's more useful than an anonymous doctor on a hotline*.

Key Informant 1

*The conversation is still much, much more important. So, we also had patients who still called us despite the negative COVID test and wanted to discuss the situation. So, I think that the digital tool (OFTT) alone doesn't really play a role with regards to fear. The conversation is much, much better here*.

Key Informant 1

*Now it's the first time that I hear about the role of the tool (OFTT) in allying fear and on anxiety. That was not the initial goal of the tool. But I think it might have these effects simply because people know exactly what they have to do after its use*.

Key Informant 5

### Medical Decision Making

All the health care providers revealed that the tool (OFTT) was useful in recommending patients to test or not to test. For pediatricians, the recommendations to test for kids differed from what the OFTT suggested and the guidelines they got from their professional body. Key informants revealed the following;

*The challenge with an OFTT in paediatrics is that it gives the parents in paediatrics a false sense of security and that is a danger because children tend to deteriorate very fast. The OFTT is good for triage, serves time in history taking but cannot replace a visit to the doctor. Every child should be seed by a paediatrician*.

Key Informant 2

*Children often present with flu like symptoms 10 to 12 times a year. How useful is an OFTT in such cases? Maybe an OFTT that helps them decide whether a visit to the grandmother is allowed; yes or no, could be more helpful*.

Key Informant 2

*But at the end, there is no medical consultation in a test centre either, but pure swabbing-test. If someone is sick enough, where a medical consultation is needed, then they end up in a separate emergency area anyway. And then you have to take the history again from the human, medical side anyway. From that point of view, I believe that such a tool (OFTT) is then superfluous in very ill patients*.

Key Informant 2

### Utility of Tool as Information Source

Health care providers revealed being lost at the beginning of the pandemic with little knowledge of the novel virus. They however commend the authorities for stepping in and helped them navigate what to do and what not to do. They revealed the following;

*I heard from people around me that they found it difficult to get reliable information on how they can be tested. And this is what is really difficult in Switzerland because it's different everywhere. And we cannot provide national information. We can only tell the people that this is the place where you will find information at the cantonal level*.

Key Informant 5

*The most challenging thing at the beginning was really to get all the information. We usually get this from the federal office of public health, of course one can visit the WHO websites or the US websites. In future, I really would like to have a tool (OFTT) for public health and epidemiology professionals to get a general overview easily. Some networks have the possibility to share information but it was really hard work to get a feeling of which direction to take at the beginning. This is just too much work for individual doctors, so such tools (OFTT) are really important*.

Key Informant 4

### Utility of Tool in Planning and Systems Thinking

The health care providers were not involved in tool (OFTT) development and also did not show a real interest in utilizing the data in planning. That could be because they are clinically focussed and not research oriented. Most of the OFTTs were a result of private or public institution initiatives. One said the following;

*We ourselves do not have the mandate nor the capacity to develop OFTTs. We collaborated with cantons that initiated OFTT -Waadt and Bern*

Key Informant 5

*Data issues are a challenge, for example there is no link between the Swiss COVID app and the OFTTs because the Swiss COVID app is a medical device*.

Key Informant 5

### Utility of Tool in Testing-Gatekeeping

Tool (OFTT) utility depends to a great deal, also on testing capacity. Other factors affected access to COVID-19 testing thereby limiting the utility of the tool.

### Infrastructural Issues

Some health care providers who had the infrastructure adapted their practices and ended up with a COVID and non-COVID entrance and consultation rooms. Those that did not have the infrastructure to test for COVID-19 found it too cumbersome and so did not provide testing. Some revealed the following;

*So, theoretically anyone can do it (testing for COVID-19). But from an organizational point of view, it is a great effort in practice, right? You have to make sure that other people don't get infected. So, we have now organized a track that we simply test patients in the evening, they have to wait outside, are brought in by the MPA and come into a room. This room will then be ventilated and will not be used any more that day. And I don't think that every practice can provide this infrastructure. Putting a symptomatic patient in a waiting room and then leaving them there with the other people is probably not the correct approach. And the effort is relatively high for the 50 Francs fee that we now get from the FOPH for each test*.

Key Informant 1

The tool utility in testing was generally perceived as good but the authorities saw room for improvement. Below is what one key informant said;

*So, the tool was not perfect because some filled the OFTT during the weekend and could only get an appointment for testing on Tuesday. There should be no delays between use of OFTT, and getting tested. Ideally one should be able to get tested around their street corner, go out, walk for 5 minutes, get tested, positive or negative and take appropriate action. So, we are working on that, to shorten this process. That is also one of the reasons we introduced the rapid tests, we want to have an answer as fast as possible. We looked at the data around two months ago. First, people wait long at home until they get the courage to test, then they are told to wait for two days before getting the testing appointment and then an additional two days before receiving results. This is the main challenge in this pandemic-time. Any tool to be developed in future, should shorten that time*.

Key Informant 5

### Potential Telehealth Stumbling Blocks-Planning and Systems Thinking

Both the health care providers and health authorities cited challenges they perceive as barriers to telehealth in general.

### Regulatory Issues

Recommendations and rules have different effects particularly in a pandemic context as revealed below;

*Clearly recommendations from health authorities are not implemented. You need the law; you can't shake it. The recommendations are nice to have but that doesn't really help in a pandemic situation, in my opinion. At the moment I have issues with decentralized cantonal government delegations. I still think it would actually be a federal matter to decide there. It is a bit of a problem if every canton to bakes its own rolls and only do it half-way*.

Key Informant 1

### Reimbursement Challenges

Some health care providers raised issues of reimbursement with regards to telehealth services (general) as revealed below;

*We did a lot of telephonic consultations during Corona, for example with children after a home accident. You don't really have to see them immediately for example if toddlers fall. And we talked a lot on the phone and said: Take a photo. So, we've already given a lot of advice on the phone, actually. But we do not do that anymore, because um... I have to say on the phone, I can't bill that. But when they come to my practice, I can bill that. The amount of work is the same. I simply do not earn with telemedicine*.

Key Informant 3

### Attitudes of Health Care Provider

Many health care providers revealed that it is challenging when patients come into the consultation with a recommendation from an online tool (OFTT). For them it is important to know the tool the patient is referring to, the source of tool, what it recommends, when and where. Some health care providers also believed that their patients are not ready for OFTTs. One participant revealed the following;

*Even if one sees the patient‘s face over a video consultation. I don't think that is comparable to physically consulting with your doctor you've known for a long time. An OFTT can‘t hold candle (is not comparable) to a doctor*.

Key Informant 1

### Doctor-Patient Relationship

Most health care providers cited the doctor -patient relationship as a critical element of their profession that telehealth cannot rival or replace. Others revealed that when they have a patient that says I used an OFTT before, then they reiterate that an online tool is an online tool and not a doctor. Below is what was said;

*Have the patient talk to a random GP after OFTT use? Well, it is probably better than nothing. But I think the family doctor who has accompanied him for years has a better role there than someone the patient does not know. It's the relationship that matters. We know the patients and their family situation; the patient doctor relationship is very important*.

Key Informant 1

### Demographic Factors of GP Population

Other health care providers cited the average age of the current GP as a stumbling block in the adoption of OFTTs and telehealth in general, as this generation did not grow up with technology. Below is what was said;

*I think it just takes more time, the older generation who grew up without computers will never learn this. And the younger ones, they will grow up with telehealth and will use it. But we still have a lot of people who grew up without computers and that's too far for them. They won't be able to adapt to such tools*.

Key Informant 1

### Pandemic Fatigue

Some health care providers expressed a desire to return to normality and see their patients face to face rather than focus on telehealth (general). Below is what was said;

*We are tired and we do hope that there will be nothing like this again for the next 20 or 30 years*.

Key Informant 3

### Potential Use of OFTTs in Future

Both health care providers and authorities cited the utility of online tools (OFTT) in education, planning and streamlining of information now and in future pandemics. Below is what was said in this regard;

*Well as far as I see this development has already started that more and more online tools (OFTTs and others) are being implemented, even apps for mobile phones. I guess they do have to play a role in the future from now on. It's not always clear what the role is exactly and neither are the rules. So, I think we need to find out who needs a tool for what, what do you want to learn or implement through the tool and then find the appropriate solutions. Up to now, not all that we saw was really thought out and useful, I think*.

Key Informant 4

*Actually, right now, we have challenges daily with really getting a grip on outbreaks in old persons homes, old peoples‘ homes have become hotspots. It would be great if telehealth could develop a tool (OFTT or other) that could assist old peoples‘ homes with infection control guidelines and outbreak prevention and management*.

Key Informant 4

*A tool that could ensure that we have sufficient PPE and material in stock. That would make things a lot easier. But also, a tool (OFTT or other) that could prepare and teach medical students simple but important things such as how to wear and remove masks, body suits and gloves and correctly*

Key Informant 1

*Even now, today I must say I find it quite challenging to get good sources of information on different levels. You want to know what is happening in Switzerland but also in Europe and then in the world again and you really have to switch from just A to Z and of course we do have access to some websites, but tools that aggregate this data a bit better would be nice to have, something official where I know there are professional teams behind the tool (OFTT or other) aggregating and putting the information together, evaluating best evidence in different contexts*.

Key Informant 4

*And then it was really clearly defined that we were really only allowed to do emergency treatment only. Exactly, clearly specified by the cantonal doctor what emergency treatment is under the threat that our professional license will be withdrawn if we do more. I had just started with the practice. My very existence was at risk. A tool (OFTT or other) to help us carry on with work and not threaten our own existence would be great*.

Key Informant 3

*Um... ultimately, it would be nice if there were a tool (OFTT), where one would simply feed in all the symptoms and then according to FOPH criteria, get yes test or no instead of clicking two or three listed symptoms as most OFTTs. It would make sense to have tools used across hospitals, across Switzerland or at least across the canton. Then at least the data would be uniform and there would be a certain continuity of care. At the moment, we still have to ask again for the symptoms again before we examine the child-a waste of time*.

Key Informant 2

## Discussion

**Figure 1:**
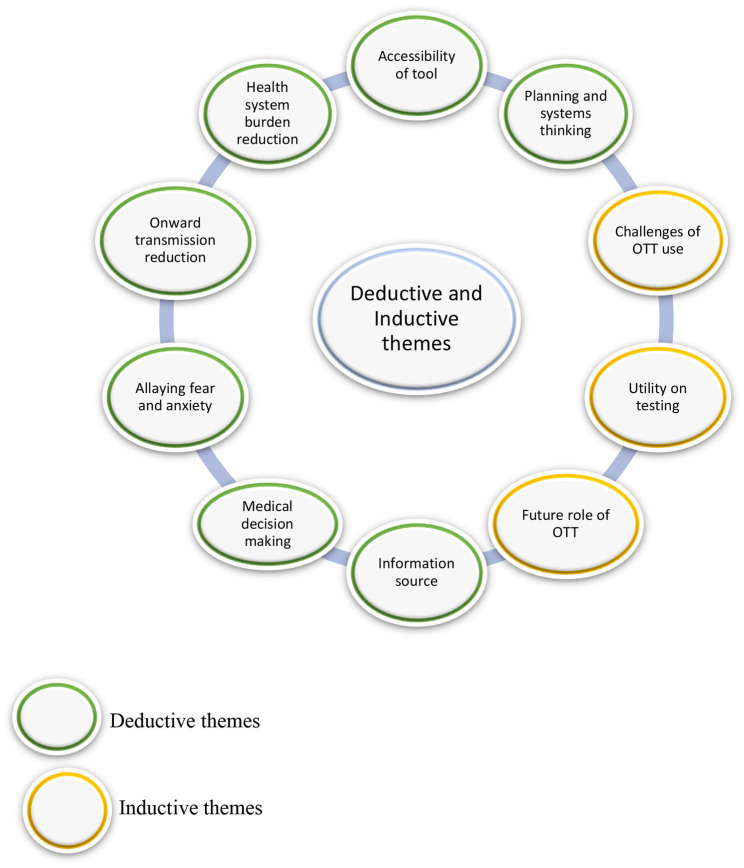
Summary of inductive and deductive themes.

This study explored the utility of a COVID-19 OFTT as perceived by health care providers and authorities. We further elaborate on potential barriers and potential role of OFTTs and telehealth (in general) in future. There were both individual and health system level benefits of the tool (OFTT). Our main findings revealed the following themes; (i) accessibility (ii) health system burden reduction (iii) utility in preventing onward forward transmission (iv) utility of tool in allaying fear and anxiety (v) utility of tool in medical decision making (vi) utility of tool as information source (vii) utility of tool in planning and systems thinking. The health care providers and health authorities further provided information on potential barriers and telehealth utility (OFTT and others) in future (see Figure 1 and Table 3).

**Table 3:** Summary of emergent themes.

**Theme**	**Category**	**Unit meaning**
Accessibility of tool	Cantonal authorities shared tool with health care workers Some practitioners shared tool link Tool source One tool for whole canton recommended Socio-economic status	- Tool link shared with health care providers - Accessible to patients from some practices - Tool not advertised - Trust depends on this - Different cantons using different tools - Low socio-economic group seems to trust medical authority
Health system burden reduction	Reduced phone calls	- Patients followed recommendations
Onward transmission reduction	Tool is good for triage of patients Ambivalence as employer	- Patients stayed at home - Tool affects staffing and service provision - Different tool for different clients?
Allaying fear and anxiety	Pediatrician sees this as his role	- Talked to the parents myself - Human factors, different parents react differently - An unintended positive consequence according to FOPH
Medical decision making	Challenges in pediatrician	- Symptoms not always clear - Clinical exam important in children
Information source	Guidance for health care providers Guidance for patients	- FOPH information, videos and charts were very useful
Future role of OTT	Infection prevention and control (IPC) tool for old peoples‘ homes Streamlining and sifting of information -FOPH OFTT for other conditions personal protective equipment use teaching Online tools for education of health care workers	- Reliant on private initiatives, commercial telemedicine providers and insurance companies-initiated activity (private vs. public sector initiatives)
Challenges of OFTT use	Cantonal medical doctors have policing role and mandate Attitudes of some health care workers physician-patient relationship Age and digital literacy of health care workers Pandemic fatigue	- Economic and existential challenges for some healthcare workers e.g., problems with charging of telehealth services - Face to face consultations nurture this - Change the generation of doctors - Health care providers are tired and want to return to normalcy and not focus on telehealth
Planning and systems thinking	Limited data access FOPH has no mandate and no capacity Legal issues	- OFTT could be useful in predicting upcoming waves - Collaborated with cantons that initiated OFTT - No link between Swiss COVID app and OFTTs and other tools in general - Reimbursement of online consultations not clear
Utility on Testing	Infrastructure issues Re-imbursement	- Some adaptations needed to be done and space challenges restricted uptake of testing - Price for testing was not attractive enough bearing in mind the adjustments and investments needed to enable this

### Accessibility

The health care providers and health authorities concur with patient perspectives (13), and unanimously agree on the importance of digital tool accessibility. Responsibility for the development and dissemination of such a tool (OFTT) emerged as an unresolved problem, especially in the Swiss setting with changing responsibilities between the FOPH and the cantonal medical offices during the pandemic. To ensure rapid and comprehensive availability of an OFTT in future pandemics, this responsibility should be clarified in advance and, if possible, preparations for its creation should be made (e.g., to address the problem of registration of OFTT as a medical device). The health authorities highlighted their limited role in digital tool development (OFTT), citing having no mandate and capacity, limiting them to making recommendations only. The initiatives to develop and advance OFTTs and other tools, remains largely in the hands of the private sector-a challenge and an opportunity. Some health care providers were very happy when the tool (OFTT) was developed and quickly shared the link to the tool on their homepages to ensure increased access. In addition to independent dissemination by health care providers on their own homepages, a dissemination strategy is needed. How to make the tool accessible to all social strata needs further exploration. Our study demonstrated that those with higher education tend to use OFTTs concurring with findings that revealed that those with high income tend to use telehealth (16). On the other hand, when it comes to following recommendations, the higher socio-economic strata tends not to follow recommendations readily (13). On the contrary, according to health care providers, those with low education seem to respect medical authority. Could that be the reason the low education group seems not keen to use OFTTs?

### Source of Tool

Trust in the source of the tool e.g., a well-known. institution emerged as very important to health care providers and authorities alike. These findings are in line with patient perspectives (13) and research from elsewhere (17). The source of the tool and the institution behind it therefore affect its adoption and utility (18). This could be another reason why the development and deployment of an OFTT should be done by trusted institutions, such as universities, or by government agencies themselves.

### Socio-Economic Status and Telehealth

Low socio-economic has been cited as a barrier to telemedicine (15). Health care providers shared insights that could shed more light on the phenomenon. According to health care providers, the low socio-economic group accepts and respects medical authority and recommendations readily. They revealed that it is the higher socio-economic group that often disregards recommendations as they have alternative sources including the internet.

Could it be that the low adoption of telehealth by the low socio-economic group is associated with the trust and respect this group has in medical authorities rather? It is worth bearing in mind that despite good intentions of policies, these might make people in the lower socio-economic group feel inferior or blamed (19) rather than understood. In summary, are we asking the right questions when it comes to low uptake of telehealth by the low-socio economic group? Stigmatization is defined as a context where a personal attribute e.g., not using telemedicine, leads to the devaluation of the person or group because of that attribute (19). Are we imposing needs and preferences (telemedicine use) the low socio-economic group does not have? In support of our findings, stigmatization of people from low socio-economic status remains a challenge (19). These findings concur with patient perspectives (13) that revealed a low uptake of telehealth in this group.

### Utility of Tool as an Information Source

Both health care providers and health authorities found the OFTT useful as an information source during the pandemic concurring with patient perspectives (13). They also reiterated the need for OFTTs in future, that would help them streamline emerging research and evidence, into information that could aid decision making. In support of our findings, telehealth has been reported as having a role in the dissemination of information (1, 3, 15, 20). Metadata generated during OFTT use could be used for development and monitoring of the impact of the recommendations, something of great importance. This is currently not being done by the health authorities in Switzerland.

### Utility in Health System Burden Reduction

All the health care providers cited that the tool reduced the high volumes of calls from concerned patients. The health authorities acknowledged the same effects. These findings concur with patient perspectives and research from elsewhere that revealed the same (13, 20, 21).

### Utility in Preventing Onward Transmission

The health care providers in their role as a doctor, found the tool effective in triaging patients and making sure that suspected COVID-19 patients received instructions from the comfort of their own homes, effectively reducing exposure and onward transmission (3, 20). Some health care providers however, demonstrated ambivalence to the OFTT utility in their role as an employer, when the person in question was a staff member. They found that the tool was indiscriminate and made recommendation to staff to stay at home, with the appearance of light symptom. This generated unintended effects like unpredictable staffing, affecting service delivery and patient outcomes. In England and Wales an NHS COVID-19 app sent self-isolation alerts to 600,000 people between 8 and 15 July causing an outcry from employers as these affected services amid serious staff shortages (21), further confirming that this issue goes beyond the health sector. In general, COVID-19 OFTTs have been found to vary greatly in sensitivity and specificity (22). In the context of a health care worker shortage, is there a need for OFTTs to be specifically designed for different target groups e.g., health care staff, kindergarten staff, food handling staff and office staff? Is there a need for sector specific OFTTs? Beyond healthcare professionals, effects of the recommendations given is important for employers, self-employed workers and policy makers as these may have an effect on adherence to OFTT recommendations.

### Utility of Tool in Allaying Fear and Anxiety

The health care providers interpretations of the utility of tool (OFTT) in this regard differed greatly from the patient perspectives (13). Some health care providers stated that allaying fear and anxiety is their role and that they did this a lot during lockdown. Pediatricians in particular, explicitly stated the need for the parents to bring in the child to the practice for the doctor to allay fear and anxiety. They cited human factors as playing a role here. According to them, different parents react differently when confronted with their own sick child (23). It is not clear if this is influenced by the education, culture, personality, previous experience as a parent or none of these. Further exploratory studies are needed in this regard. Interestingly, for the health authorities, the utility of the OFTT in allaying fear and anxiety emerged as an unintended effect (24), that came as a pleasant surprise. The health authorities however, demonstrated a growing interest in the utility of the tool in allaying fear and anxiety, as more neuro psychological effects of COVID-19 emerge as a public health concern. In support of our findings telehealth has been revealed to allay fear and anxiety during this pandemic (15, 25–28). Additional research is needed to bridge the gap between the patients' and health care providers' perceived utility of an OFTT in this regard.

### Utility of Tool in Medical Decision Making

Many health care providers repeatedly highlighted the limitations of an online tool (OFTT) recommendation since in their experience, recommendations for example test, are not usually followed. They suggest that rules work better. Contrary to provider perspectives, the patients themselves found the OFTT very useful in decision making (13). From the pediatrician's point of view, children have unclear symptoms and tend to deteriorate fast. A physical presentation of a child to the physician is important, concurring with findings elsewhere (9, 29, 30). This danger must be considered when developing an OFTT for children.

### Potential Barriers to Telehealth

Barriers to telehealth have been identified as patients, staff, team, business, financial as well as governance and regulatory related barriers (10).

#### Utility of Tool in Planning and Systems Thinking

As alluded to earlier, the health authorities cited data access issues as the FOPH has no mandate and also limited capacity in setting up OFTTs as well as evaluating the generated data. For future pandemics, the structures, including personnel and infrastructure, for evaluating and using the data would be useful.

#### Doctor-Patient Relationship

The doctor patient relationship is one core element that has been attributed to the success of the GP model in Switzerland. Many health care providers were concerned with the way telehealth including OFTTs could affect the doctor-patient relationship. For many health care providers, patient doctor relationship was revealed as tantamount to quality of care and the issue of medical liability remains unresolved (4, 31). That raises a number of questions; is the doctor patient relationship subject to evolution? Is it possible to start a doctor patient relationship online or is it only feasible to maintain one? Concurring with our findings, concerns have been raised elsewhere that telehealth could fundamentally alter the personal face to face doctor patient relationship, a model that has been used for generations (4, 30). Is telehealth disrupting tradition? (30).

#### Health Care Provider Attitudes

In support of our findings, staff barriers to telehealth have been reported elsewhere (4). Some suggested that OFTTs, tele or video consultations are suitable for triage and follow up, but not initial consultation and diagnosis. According to literature (30), the first visit must be in person, as neither physical examination nor anamnesis can be eliminated, concurring with our findings.

The health care provider themselves seem not involved or at most in the fringes when telehealth approaches including OFTTs are implemented. The lack of their involvement and consultation might be a contributing factor to their lack of enthusiasm. Involvement of these local health care providers in future is critical for the uptake of telehealth (24, 32). Elsewhere, clinician attitudes and acceptance of telehealth have been associated with telehealth uptake and sustainability (12), concurring with our study. Is it possible that health care provider attitudes to telehealth are also standing in the way? According to one study (33), 65–80% of patient that use telehealth before an emergency consultation, do not communicate this to the attending emergency physician (17). Why is it so? The attitudes of health care professionals influence new technology uptake (9). Could it be both-lack of involvement and attitudes?

#### Lack of Technical Affinity and Resistance to Change

Another staff variable that got revealed as a potential barrier to telehealth was age. Change is pervasive and brings about uncertainty, hence humans put up resistance (34). The average GP in Bern grew up without most of the technology available today thereby falling into the group known in some contexts as born before technology (BBT) or technophobes (35). According to some healthcare providers, the new generation of younger doctors that have grown up with the technology, could increase the acceptance of telehealth. In line with our findings, staff, behavioral, technical, clinical, and organizational issues have been reported elsewhere as potential stumbling blocks to OFTTs and telehealth in general (4).

#### Data Privacy and Regulatory Issues

Data privacy, regulatory and telehealth tariffs emerged as potential barriers for all health care professionals, particularly dentists who do not get reimbursed for telehealth consultations. In line with our findings, financial and clinical justification for telehealth investment has been identified as a potential barrier (4). Though our findings are mostly positive, it is worth highlighting that telehealth including OFTTs have also been associated with some negative effects (36). For example, a systematic review about digital interventions to help patients self-care didn't find any reduction in usage of urgent-care services, maybe because of the lack of involvement of patients in intervention development and perceived difficulties in app usage (37). Data privacy and regulatory issues also emerged as stumbling blocks in the COVID-19 OFTT utility in planning and systems thinking (34).

### Future Telehealth Opportunities

Both health care providers and authorities expressed the need for future telehealth tools including OFTTS, that could assist health care workers and decision makers in sorting out relevant, best evidence on prevention, treatment, care as well as behavioral actions needed to reduce transmission in a pandemic setting. Authorities see the potential of telehealth in developing tools including OFTTs that could help generate real time guidelines according to best evidence in a pandemic setting. On the other hand, the health care providers and authorities both cited their limited mandates and capacity in future development of OFTTs. Collaboration with Universities and institutions could further strengthen the roles of authorities in telehealth development in future.

## Strengths and Limitations

Our study explored health care provider and authority perspectives on the utility of a COVID-19 OFTT. Our study provides the rare insights into discussions held with health care authorities at different levels in Switzerland. The attitudes of these actors affect the adoption of telehealth including OFTTs. The provider and authority perspectives could be compared with patient perspectives, providing a unique data source triangulation opportunity.

One shortcoming might be our sample size. Only 5 key informants could be interviewed. In line with qualitative study designs data saturation and rich narratives were reached after the third interview. In future, focus group discussion with all stakeholders could foster dialogue and collaboration by bringing the different parties together, potentially moving telehealth forward.

## Conclusion

Both the health care providers and authorities perceive the COVID-19 OFTT utility to be multifaced. There were both individual and health system level benefits of the tool. Once the access issues had been resolved, the OFTT was perceived as useful in health system burden reduction, onward forward transmission prevention, medical decision making, and as an information source. The utility of the OFTT in allaying fear and anxiety was explicitly revealed by patients and less so by health care providers who viewed allying fear and anxiety as their role rather. The health authorities were interested in the utility of the tool in this regard and are looking forward to more evidence as COVID-19 mental health issues become a public health concern. All in all, telehealth has the potential to influence both the actual and perceived quality of care (4) depending on which side of the divide one is on, as a health care professional, patient or authority. Data privacy, lack of systems thinking in planning and regulatory issues seem to stand in the way of the COVID-19 tool utility. In general, telehealth is seen as having the potential to develop tools, particularly OFTTs that could help produce real-time guidelines according to the best evidence in pandemic settings.

## Data Availability Statement

The original contributions presented in the study are included in the article/Supplementary Material, further inquiries can be directed to the corresponding authors.

## Ethics Statement

This study is embedded in an online forward triage tool set up by the Insel University Hospital in a pandemic setting to primarily prevent health system over load. The evaluation of the usefulness of this tool to the health stake holders, patients, health care providers and health authorities was deemed as quality evaluation hence the Ethics Committee of the Canton of Bern, Switzerland waived the need for full ethical review (Req-2020-00289) on the 23rd of March 2020 and granted us permission to carry out the study. Written informed consent was obtained from all participants or their legal guardians to participate in the study.

## Author Contributions

JM, TK, AM, MM, WH, SH, and TS: study design and idea. JM, AM, and TS: qualitative data analysis. JM and TS: writing of first draft. JM, TK, AM, MM, WH, and TS: revision of final draft and approval. TS and WH: project administrators. All authors contributed to the article and approved the submitted version.

## Funding

This work was supported by Swiss National Science Foundation (Project ID: 196615). The funder has no influence on the content of the manuscript or decision to publish it.

## Conflict of Interest

TS holds the endowed professorship for emergency telemedicine at University of Bern, Switzerland. The funder, Touring Club Switzerland, has no influence on the research performed, the content of any manuscript or any decision to publish. WH has received research funding from the European Union, the Swiss National Science Foundation, Zoll Foundation, Drr Medical Germany, Mundipharma Research UK, MDI International Australia, Roche Diagnostics Germany; provided paid consultancies to AO Foundation Switzerland and MDI International Australia; received financial support for a congress he chaired from EBSCO Germany, Isabel Healthcare UK, Mundipharma Medical Switzerland, VisualDx USA, all outside the submitted work. The remaining authors declare that the research was conducted in the absence of any commercial or financial relationships that could be construed as a potential conflict of interest.

## Publisher's Note

All claims expressed in this article are solely those of the authors and do not necessarily represent those of their affiliated organizations, or those of the publisher, the editors and the reviewers. Any product that may be evaluated in this article, or claim that may be made by its manufacturer, is not guaranteed or endorsed by the publisher.

## Abbreviations

OFTT: Online Forward Triage Tool
FOPH: Federal Office of Public health
GP: General Practitioner.
