# Supplementary material for: The Utility of an Online Forward Triage Tool During the SARS-CoV-2 Pandemic: Health Care Provider and Health Authority Perspectives
Source: Front Public Health. 2022 Jul 8;10:845996. doi: 10.3389/fpubh.2022.845996 (PMC9305458; doi:10.3389/fpubh.2022.845996)
Supplement: Supplementary file 1 [file Data_Sheet_1.pdf]

Appendix 1: Interview Guide: Coronatest.ch -Health Care Providers

Rapport

0Please describe what your role (organisation) was in the COVID-19 pandemic (formal, informal, evolved, new roles emerging)

Utility in reducing Health system burden

- Did you refer any patients to our online tool coronatest.ch during the pandemic? Why did you do that or why not, other tools used, which and why?
- What attributes should a tool have to be useful to you and your practice? Information, utility, what did patients look for?
- Looking back did you respond accordingly or did the pandemic take you by surprise? (Dismissed patients as having hysteria)

Utility as a reliable information source

- What do you recommend to make the coronatest.ch more effective-suggestions for improvement? How did you keep yourself up to date?

Illness and testing Experience

- What was your own COVID-19 experience that you would like to share (prompt falling ill, fear of falling ill, protecting oneself, protecting other patients, challenges- testing kit availability, did you offer testing, why and why not, comments to changing testing criteria)?

Challenges and Recommendations

- Any lessons you learnt at personal or organizational or health system level
- What was the greatest challenge you experienced?
- How do you plan to deal with this in future pandemics?
- How best should such an epidemic be handled in future
- Adequacy of medical education in such situations and recommendations
