# Supplementary material for: The Utility of an Online Forward Triage Tool During the SARS-CoV-2 Pandemic: Health Care Provider and Health Authority Perspectives
Source: Front Public Health. 2022 Jul 8;10:845996. doi: 10.3389/fpubh.2022.845996 (PMC9305458; doi:10.3389/fpubh.2022.845996)
Supplement: Supplementary file 2 [file Data_Sheet_2.pdf]

Appendix 2: Interview Guide: Health Authorities

Rapport

0Please describe what your role (organisation) was in the COVID-19 pandemic (formal, informal, evolved, new roles emerging)

Utility in reducing Health system burden

- Did you come up with interventions to reduce the health system burden e.g., online tool? Why did you do that? (Public demand, duty), tool experience-what worked well and what did not?
- Looking back at the pandemic, what had BAG planned earlier (pandemic plans) what did they plan well, and what aspects of the pandemic took BAG by surprise? (PPE, testing kits, capacity, demand for information from health care professionals and from the public)
- Did BAG use coronatest.ch? What did you like and what did you find deficient?
- BAG developed an OFTT. What prompted you to do so? (Public demand, available tools were not good enough etc.). Did you advertise the tool? -what can be done to ensure all segments of the population have access? How do you use the tool as an organization? (pattern and trend recognition- (who uses tool, from where, why, how often), feedback loop, tool improvement-how often and how is the data collected? Is it used in planning and resource allocation and to detect outbreaks?

Utility as a reliable information source

- How did you keep your OFTT up to date with regard to information (evolving evidence)? Who does that, how, and how often?
- Questions about OFTTs in general: What attributes does an OFTT need to have from BAGs point of view- accessibility, information source, reducing health system burden, allaying fear and anxiety etc? In light of the boom of OFTTs, does BAG play a role in making sure the Swiss public only uses reliable, validated OFTTs?
- In preparation for future pandemics-are there plans to develop, strengthen and expand OFTTS tools? (To include other conditions etc)

Illness and testing Experience

- What was your own COVID-19 experience that you would like to share (prompt falling ill, fear of falling ill, protecting oneself, protecting others at work place and home, challenges?

Challenges and Recommendations

- What was the greatest challenge you experienced? (Prompt dealing with public, media and fake news)

- Any lessons you learnt at health system level? (What does BAG expect from the health care system in case of a pandemic and online tool use-hospitals vs ambulatory care)

- From your pandemic experience what do you think of the Swiss medical education? (Is it adequate, adaptations needed, recommendations)
